# Psychological distress, emotion regulation, neuroticism, and sexual relationship on patients with temporary ejaculation failure *in vitro* fertilization-embryo transfer treatment

**DOI:** 10.3389/fpsyg.2022.1090244

**Published:** 2023-01-06

**Authors:** Xinting Zhang, Lexia Yang, Wei Wang, Lejin Yang

**Affiliations:** ^1^Department of Pediatrics, Qilu Hospital of Shandong University, Jinan, China; ^2^Nursing Department, The Third Hospital of Jinan, Jinan, China; ^3^Department of Psychology, Qilu Hospital of Shandong University, Jinan, China

**Keywords:** temporary ejaculation disorder, psychological distress, neuroticism, cognitive reappraisal, sexual relationship

## Abstract

**Objective:**

Temporary ejaculation failure on the oocyte retrieval day might leading interruption of the oocyte retrieval procedure. The present study aims to understand the psychosocial factor that affects men with temporary ejaculation failure (TEF) *in Vitro* fertilization-embryo transfer (IVF-ET) patients, and thus provide new ideas for optimal clinical treatment.

**Study design:**

In a prospective study, the male patients during IVF treatment in a reproductive center of a tertiary hospital in Shandong were divided into two groups, 70 men with TEF and 79 normal controls. General population sociology and clinical disease were investigated, and the Kessler 10 scale, emotion regulation questionnaire, big five inventory questionnaire, and sex subscale of marriage quality were used to assess the psychological distress, emotion regulation, neuroticism, and satisfaction with sexual life.

**Results:**

The scores of perceived distress and neuroticism of the TEF group were higher than the non-TEF group (*p* < 0.001), and cognitive reappraisal and sexual relationship were significantly lower than those in the non-TEF group (*p* < 0.001). Psychological distress (OR 1.130, *p* = 0.031) and neuroticism (OR 1.096, *p* = 0.050) were risk factors for TEF, while cognitive reappraisal (OR 0.883, *p* = 0.004) and sexual relationship (OR 0.712, *p* < 0.001) was protective factors.

**Conclusion:**

The present study demonstrates that psychosocial factors influence TEF in IVF-ET patients, which provides the basis for the prevention of the occurrence of TEF in a male undergoing IVF-ET.

## 1. Introduction

*In Vitro* fertilization-embryo transfer (IVF-ET) technology has been widely used as an assisted reproductive technology for the treatment of infertility. In particular, among the men with normal sexual function and routine insemination who received IVF-ET treatment, nearly 10% of men will experience temporary ejaculation failure (TEF) during the artificial insemination cycle of the husband’s sperm (Glina et al., 2005), failing to obtain sperm which affect the normal treatment process. TEF was considered an unexpected ejaculation failure that could not obtain sperm samples by masturbation (Lu et al., 2009; Ozer et al., 2020). Failure to provide a sperm sample on the day of the oocyte collection could put the couple at risk of having the oocyte collection canceled or the oocyte frozen, or even their efforts of the previous months in vain (Junsheng et al., 2013; Ozer et al., 2020). The occurrence of TEF is mostly affected by living habits, the environment for obtaining sperm, and a variety of psychological factors (Lu et al., 2009; Wang and Xue, 2021; Xue and Wang, 2021). Previous studies have indicated that men undergoing IVF-ET treatment tend to have concerns about abnormal semen results and the outcome of artificial insemination treatment with sperm, and even develop mental illnesses such as anxiety and depression (Peronace et al., 2007). The phenomenon is very common and evident in men undergoing treatment with infertility (Folkvord et al., 2005). The failure rate of sperm retrieval during husbands’ artificial insemination cycles is significantly higher than outpatient semen analysis, suggesting that IVF-ET treatment can put patients under ideological pressure. In addition, behavioral therapies to reduce stress have been shown to improve IVF outcomes (de Liz and Strauss, 2005). However, few researchers focus on TEF and its influencing factors in men with normal sexual function but suffering from TEF.

Among the various factors that affect the levels of psychological distress during treatment, emotion regulation strategies, personality, and sexual satisfaction are important psychosocial factors (Preece et al., 2020). Emotion regulation was regarded to manage the emotional states which are considered a key factor in psychological issues (Ma and Fang, 2019). Based on Gross’s process model of emotion regulation, there are two regulation strategies: Cognitive reappraisal, where a person attempts to change how he or she thinks about a situation to change its emotional impact, and expressive suppression, where a person attempts to inhibit the behavioral expression of his or her emotions (Preece et al., 2020). When someone successfully uses cognitive reappraisal to regulate their emotions, they report higher levels of self-esteem and life satisfaction (Brewer et al., 2016), but unsuccessful use of this technique was linked to social anxiety (Kivity and Huppert, 2016; Kivity et al., 2021). In addition, neuroticism, as one of the personality characteristics, is commonly defined as the tendency to have a negative effect, particularly when a person is threatened, frustrated, or confronting loss, and it is believed to be rather stable over time (Pereira-Morales et al., 2019). Neurotic people had more daily troubles, more severe emotions, and stronger reactivity to recurring problems, which may predispose them to depression and anxiety (Deimling et al., 2017; Mommersteeg et al., 2017). Moreover, as relationship satisfaction, sexual satisfaction is a critical indicator of sexual health, which is a two-dimensional notion of having a personal perspective and dyadic processes related to a close relationship (Nakić Radoš et al., 2022). Plenty of researchers have indicated that higher levels of sexual satisfaction could reduce the rate of ejection disorders. For instance, men with delayed ejection report lower levels of sexual dissatisfaction (Chen, 2016). Moreover, low sexual satisfaction was reported after the infertility diagnosis which indicated infertility status and its related psychological concerns can underlie sexual dysfunction (Lotti and Maggi, 2018). Thus, the present study aimed to investigate sexual relationship on the occurrence of TEF.

In summary, the present study aims to explore the relationship between psychological distress, emotion regulation, personality characteristics, sexual relationship, and occurrence of TEF during IVF-ET treatment, to provide the basis for effective clinical interventions and prevention. According to the previous studies (Allen and Walter, 2018; Maestre-Lorén et al., 2021; Fischer et al., 2022), the study proposed that psychological distress and personality of neuroticism could induce the occurrence of TEF and better emotion regulation and sexual relationship could prevent the occurrence of TEF.

## 2. Materials and methods

### 2.1. Research subjects

This prospective study was conducted in the reproductive center of a tertiary hospital in Shandong Province from January 2020 to January 2021 and undergoing IVF treatment. The participants were recruited from male patients with IVF treatment in the hospital. The purpose, significance, and filling requirements of the questionnaire survey are explained to them by the surveyors with standardized training. There was no reward for participation. After the participants signed the informed consent, the participant completed the paper questionnaire in the reception room and on the day after the male sperm collection. The process was voluntary and anonymous and Patient No. was filled to recognize the patients, and a single-blind technique was applied to the physicians. The sample included 70 TEF patients and 79 non-TEF patients finally, with a response rate of 82.8%. Patients with TEF were diagnosed by two physicians who completed a standard evaluation.: (1) Patients with delayed ejaculation when had sufficient sexual excitement, sexual stimulation, and strong erection for more than 30 min but still could not complete ejaculation. (2) Patients with insufficient ejaculation when a final semen volume of fewer than 0.5 ml (without losing any) after ejaculation.

Inclusion criteria for TEF patients: (1) men who received IVF treatment and have a normal sexual function; (2) men who had temporary ejaculation disorder on the day of sperm retrieval; (3) men who had no physical mental disorder; (4) men had no major life events in the past; and (5) men can understand the questionnaire, and willingly participate with informed consent. Inclusion criteria for non-TEF patients: (1) men who received IVF treatment and have a normal sexual function; (2) men who ejaculation successfully for the first time on the day of sperm retrieval; (3) men who had no physical mental disorder; (4) men had no major life events in the past; and (5) men can understand the questionnaire, and willingly participate with informed consent.

Exclusion criteria: (1) Men with congenital malformations and dysplasia of reproductive organs that were able to be identified by history taking, testicular ultrasound, and physical examination with physicians; (2) men with erectile dysfunction caused by trauma; (3) men who have a history of sexual dysfunction (premature ejaculation, erectile dysfunction, delayed ejaculation, retrograde ejaculation, semen Volume < 0.5 ml); (4) men who failed to put semen into a sterile semen cup or spill part of semen outside; (5) men with a history of mental illness and taking psychotropic drugs (including antidepressants); (6) men with a history of chronic diseases and need to take hormone drugs and antihypertensive drugs; and (7) men with a history of other serious diseases. A questionnaire survey was conducted after the collection of sperm was completed on the day of female oocyte retrieval. The questionnaire survey included basic information (including age, height, occupation, residence, education, job occupation, infertility years, and treatment duration), a perceived stress scale, an emotion regulation questionnaire, a neurotic dimension, and sexual relationship questionnaire. All questionnaire surveyors have undergone training. This study was approved by the Ethics Committee of Shandong University.

### 2.2. Methods

#### 2.2.1. General information survey form

The general information survey form was designed by the researcher, including general population sociology, such as age, height, occupation, residence, education, job occupation, having children or not, infertility years, treatment duration, and masturbation history. Items examples: “What is your age,” “Do you have children or not,” and “How long have you been treating infertility?”

#### 2.2.2. Kessler10 (The 10-item Kessler psychological distress scale, K10)

The study adopted the Chinese version of Kessler10 revised by Zhou et al. (2009), with a total of 10 items and uses a 5-level scoring method (1 = “none of the time” to 5 = “all of the time”). The Kessler 10 measures the client’s psychological distress levels. The total score ranged from 10 to 50 points, and the higher the score, the more serious the psychological distress. Items examples: “Feel tired out for no good reason” and “Feel nervous.” The Cronbach’s α coefficient of the scale was 0.93.

#### 2.2.3. Emotion Regulation Questionnaire (ERQ)

The Emotion Regulation Questionnaire (ERQ) consists of two subscales, cognitive reappraisal and expression inhibition which was compiled by Gross and John (2003), and the study used the Chinese version of ERQ (Wang et al., 2022). The ERQ was a widely used measure of two emotion regulation strategies, including cognitive reappraisal and expressive suppression. The questionnaire has 10 items in total and used a 5-point scale (1 = “almost never” to 5 = “almost always”). Items examples: “I do not express my emotions” and “The way I control my emotions is just not expressing them.” In this study, Cronbach’s alphas of cognitive reappraisal and expressive suppression subscales were 0.81 and 0.68, respectively.

#### 2.2.4. The Big five inventory (BFI)-neurotic dimension

The original scale of BFI was compiled by John and Srivastava (1999) and the Chinese version was applied in the study (Zhang and Zheng, 2005), with 44 items in total. In this study, only the neurotic personality subscale was used which measured an individual on the Big Five Factors (dimensions) of personality of neuroticism vs. emotional stability. The scale uses a 5-level score (1 = “Strongly disagree;” to 5 = “Strongly agree”), and each dimension contains reverse scoring items. Items examples: “Is depressed, blue,” “Can be moody.,” and its Cronbach’s α coefficient was 0.83.

#### 2.2.5. The ENRICH marital inventory-sex life dimension

The ENRICH marital inventory was compiled by Olson et al. with 124 items in total and 12 subscales (Fowers and Olson, 1989), and a Chinese revised version was applied in the study (Li, 1999). In this study, only the sex life dimension subscale was used. The subscale measures a feeling about the affectional and sexual relationship which reflects attitudes about sexual matters, sexual behavior, sexual fidelity, and birth control (Fowers and Olson, 1989). As a Likert scale (1 = “this is true” to 5 = “it is not the case”), it has adequate validity and reliability in a clinical study. In this study, only the “sexual relationship” not the entire scale was used in this study (including 10 items). Items examples: “Talking about sex with my spouse is easy and relaxing for me,” “Sometimes, I worry that my spouse will want to seek sex outside of marriage.” And its Cronbach’s α coefficient was 0.60.

### 2.3. Statistical analysis

The SPSS 24.0 software was used for statistical analysis. The measurement data are expressed by the mean ± standard deviation, and the counting data are expressed by the frequency (percentage). Independent sample *t*-test and Chi-square test were used to analyze the scores and differences of social demographic factors, clinical data, psychological distress, emotion regulation, neuroticism, and sexual relationship of patients with TEF. Univariate or multivariate logistic regression was used to clarify the influencing factors of TEF. The results were considered statistically significant if *p* < 0.05.

## 3. Results

### 3.1. Comparison of sociodemographic factors and clinical data between the TEF and non-TEF groups

The mean age was 35.426 (SD = 6.521), BMI was 35.426 (SD = 6.521), and mostly had a history of infertility for 1–21 years, and with 1–10 years of marriage. As shown in Table 1, there was no statistical difference between the TEF group and non-TEF group in most demographic characteristics and clinical data (including age, BMI, residence, educational level, job occupation, have children or not, infertility years, treatment durations, and masturbation history; *p* > 0.05).

**Table 1:** Comparison of sociodemographic factors and clinical data between two groups.

Item	TEF group (*n* = 70)	Non-TEF group (*n* = 79)	*t*/X^2^	*p* value	Cohen’s *d* / Contingency coefficient
Age	36.77 ± 7.163	36.25 ± 6.358	−0.468	0.152	0.077
BMI	24.98 ± 3.177	25.29 ± 3.057	0.602	0.892	0.099
Residence			0.089	0.766	0.039
Urban	48	57			
Rural	22	22			
Education Level			5.030	0.081	0.181
Junior high school and below	26	38			
High school or technical secondary school	15	7			
College degree and above	29	34			
Job occupation			1.975	0.853	0.114
Manager	17	13			
Staff	28	34			
Intellectuals	5	6			
Farmers	6	9			
Freelance	12	13			
Other	2	4			
Have children or not			0.425	0.514	0.067
Have children	29	38			
Have no children	41	41			
Infertility			2.733	0.255	0.134
< 3 years	36	50			
3 ~ 5 years	22	16			
> 5 years	12	13			
Treatment Duration			0.985	0.611	0.081
< 2 years	33	40			
2 ~ 5 years	18	15			
> 5 years	19	24			
Masturbation history			5.862	0.053	0.195
Never	16	23			
Seldom	47	55			
Often	7	1			

TEF: temporary ejaculation failure, BMI: body mass index.

### 3.2. Comparison of the psychosocial factors for the TEF and non-TEF groups

As shown in Table 2, the results indicated that psychological distress, cognitive reappraisal, neurotic dimension, and sexual relationship were significantly different between the TEF group and the non-TEF group (*p* < 0.001, *p* < 0.001, *p* < 0.001, *p* =0.013, respectively), while the expression inhibition was not significant between two groups (*p* = 0.751). The scores of psychological distress and neuroticism were higher in the TEF group than in the non-TEF group. And the scores of cognitive reappraisal and sexual relationship were lower than that of the non-TEF group.

**Table 2:** Comparison of the psychosocial factors for the two groups.

Item	TEF group (n = 70)	Non-TEF group (n = 79)	*t*	*P* value	Cohen’s *d*
Cognitive reappraisal	27.63 ± 7.458	33.56 ± 5.361	5.616	<0.001	0.922
Expression inhibition	15.60 ± 5.072	15.87 ± 5.396	0.318	0.751	0.052
Psychological distress	22.71 ± 8.448	16.18 ± 3.385	−6.330	<0.001	1.039
Neuroticism	22.59 ± 6.682	16.23 ± 4.270	−6.998	<0.001	1.149
Sexual relationship	17.34 ± 3.985	22.04 ± 2.677	−2.506	0.013	1.399

TEF: temporary ejaculation failure.

### 3.3. Single-factor logistic regression analysis of TEF predictors

As shown in Table 3, the occurrence of TEF as the dependent variable and the regression equation for single-factor logistic regression analysis was used. The cognitive reappraisal, psychological distress, neuroticism, and sexual relationship were taken as independent variables. Among them, psychological distress (*p* < 0.001, OR = 1.237) and neuroticism (*p* < 0.001, OR = 1.223) were risk factors for TEF. On the other hand, cognitive reappraisal (*p* < 0.001, OR = 0.860) and sexual relationship (*p* < 0.001, OR = 0.660) were the protective factors of TEF.

**Table 3:** Single-factor logistic regression analysis of TEF predictors.

Item	*B*	S.E.	Wald	*p*	OR	95%CI	Nagelkerke *R*^2^
Cognitive reappraisal	−0.151	0.033	21.286	<0.001	0.860	(0.806, 0.917)	0.239
Expression inhibition	−0.010	0.032	0.102	0.749	0.990	(0.931, 1.053)	0.001
Psychological distress	0.212	0.044	23.812	<0.001	1.237	(1.135, 1.347)	0.316
Neuroticism	0.202	0.037	29.049	<0.001	1.223	(1.137, 1.317)	0.326
Sexual relationship	−0.416	0.071	34.191	<0.001	0.660	(0.574, 0.758)	0.434

TEF: temporary ejaculation failure.

### 3.4. Multivariate logistic regression analysis of TEF predictors

To analyze the joint effects of the various factors of TEF, the statistical indicators in univariate analysis were included in the multivariate logistic regression analysis. The results indicated that cognitive reappraisal, psychological distress, neuroticism, and sexual relationship together affect TEF. Psychological distress (*p* = 0.031, OR = 1.130) and neuroticism (*p* = 0.050, OR = 1.096) were risk factors for the occurrence of TEF, while cognitive reappraisal (*p* = 0.004, OR = 0.883) and sexual relationship (*p* < 0.001, OR = 0.712) were protective factors for the occurrence of TEF (Table 4).

**Table 4:** Multivariate logistic regression analysis of TEF predictors.

Item	*B*	S.E.	Wald	*p*	OR	95%CI	Nagelkerke *R*^2^
Cognitive reappraisal	−0.125	0.043	8.398	0.004	0.883	(0.812, 0.960)	0.595
Psychological distress	0.122	0.057	4.637	0.031	1.130	(1.011, 1.262)	
Neuroticism	0.091	0.047	3.826	0.050	1.096	(1.000, 1.201)	
Sexual relationship	−0.340	0.087	15.332	<0.001	0.712	(0.600, 0.844)	

## 4. Discussion

This study focused on the male who developed TEF and had normal sexual function during IVF-ET treatment and analyzed the psychosocial factors that affect TEF. The results suggested that psychological distress, emotion regulation, neuroticism, and sexual relationship may influence the occurrence of TEF. Accordingly, targeted clinical assessment and effective psychological intervention in the early stage of treatment can reduce the risk of TEF in men undergoing IVF treatment, thereby reducing the harmful factors affecting IVF-ET treatment.

Unlike the previous study (Wang and Xue, 2021), the present study did not find that the TEF group and the non-TEF group had a differentiated background of demographic sociological variables, such as age, occupation, educational levels, and clinical states. The reason may be related to the small sample size. Furthermore, the results indicated that men with TEF had higher psychological distress scores than those without TEF, and the former had even reached a moderate level of psychological distress. Previous research has confirmed a strong link between male infertility and psychological distress (El Kissi et al., 2013). Patients with infertility often experience a wide range of mental distress, including anxiety, depression, stigma, social avoidance, and distress, which severely reduces the quality of life in infertility patients (Zhao et al., 2022). Long-term high psychological distress levels tend to produce more negative emotions, which may reduce men’s sexual desire by stimulating the secretion of prolactin (Wang and Xue, 2021). Consistent with a previous study (Junsheng et al., 2013), the present study indicated that psychological distress could predicate the occurrence of TEF, the reason was that distress is thought relevant to sexual disorders or elevated prolactin levels (Fiala et al., 2022). Thus, applying the intervention such as yoga therapy and self-administered mindfulness reduced the psychological distress (Bai et al., 2019; Dumbala et al., 2020), which was a critical benefit for patients with assisted reproduction.

The hierarchical regression analysis indicated that underlying control for the meaningful demographic and sociological variables in the univariate analysis, Cognitive reappraisal was a critical factor which affect the occurrence of TEF. Individuals who tend to use the cognitive reappraisal have greater positive emotion experience and expression behavior, lower negative emotion experience, and less negative emotion expression behavior, while individuals who are prone to expression inhibition have less positive emotional experiences and behaviors (Gross, 2002). Cognitive reappraisal is beneficial because it helps us detect when our thoughts have shifted into a negative mode and allow us to downregulate and transform negative emotions on time (Jamieson et al., 2012). Conversely, there is no agreement on the impact of expression inhibition on emotional experience and poor psychosocial fitness. Although expression inhibition reduces an individual’s behavioral expression of emotions, it also causes amplification of physiological responses. These inconsistencies are thought to be related to the intensity of emotional stimuli (Coifman et al., 2007). Consistent with previous findings, this study found that cognitive reappraisal, rather than expression inhibition, is associated with lower distress, better adjustment, and greater well-being (Benyamini et al., 2008; Gourounti et al., 2012). Furthermore, clinical studies also found that patients are more likely to have negative cognition-induced adverse emotions during IVF-ET treatment (Dong et al., 2013; Ni et al., 2021). Various studies indicated that vulnerable groups with positive emotion regulation are less likely to have psychological distress (Gyasi, 2019; Tian et al., 2021). The current study indicated that cognitive reappraisal is a protective factor for the occurrence of TEF, which is consistent with the study that negative emotion regulation leads to anxious apprehension and further avoidance of sexual life (Stephenson and Welch, 2020). While, the psychological counseling and guidance programs may include developing positive emotion regulation strategies and regulating coping styles, which may through reduce the psychological stress and anxiety in the process of obtain sperm.

In addition, neurotic personality also has a predictive effect on TEF occurrence (Hosseini et al., 2021). Neurotic people are prone to illogical thinking, poor impulse control, and poor stress management, consistent with the previous studies, people with neurotic personalities have higher levels of psychological distress and are more likely to suffer from common mental illnesses (Jeronimus et al., 2014, 2016). In addition, people with a neurotic personality were more susceptible to negative emotions (Jeronimus et al., 2014), are less resilient to stress, and tend to engage in negative coping strategies in response to stress (Afshar et al., 2015). Facing various pressures caused by infertility, these people are more likely to produce and accumulate negative emotions such as anxiety and depression (Maroufizadeh et al., 2015). The present study indicated that neurotic personality may influence the normal process in the sexual function of men, which is consistent with the research that sexual dysfunction is accompanied with a higher score of neuroticism (Hosseini et al., 2021). We speculated that the psychological distress may mediate the association between neuroticism and sexual dysfunction. However, this speculation still needs verifications by future investigations with a specific design and mediation effect analysis.

A previous study found strong positive associations between sexual (dis)satisfaction and sexual and relational worries associated with infertility (Luk and Loke, 2019). The current study also indicated that sexual relationship affects the occurrence of TEF. A previous study has found that women who experienced greater emotional stressors associated with infertility were related to lower sexual satisfaction with their partner (Amiri et al., 2021). While the discordant sexual relationship between husband and wife caused by long-term infertility could induce ejaculation disorders in men. Under the pressure of continuous pregnancy failure, they gradually lose interest in sex and continue to accumulate negative emotions (Jain et al., 2000). Men undergoing IVF treatment could receive various pressures from themselves and society, and long-term pressure affects normal sex, which aggravates the reduce sexual satisfaction. Moreover, a previous study has applied logistic regression analysis indicated that depression, anxiety, and sexual relationship were independent predictors of the IVF outcome (Chen et al., 2016), which could be the men’s psychological distress may adversely influence the quality of their semen in infertile couples undergoing IVF (Quant et al., 2013). The present study indicated that the sexual relationship was risk factor for the occurrence of TEF, and it is necessary to pay attention to sexual relationship and provide psychological intervention to male patients undergoing IVF treatment.

This study focused on men who received IVF-ET treatment and developed TEF, analyzed the psychosocial factors, and aimed to provide a basis for reducing the TEF of such people. In the process of IVF-ET treatment, medical staff pay more attention to individual emotion regulation, personality and sexual relationship could be an effective way to promote successful ejaculation. In the future clinical implications, the questionnaires on the psychological distress, cognitive regulations, neuroticism, and sexual relationship could be applied in the patients before the sperm extraction, while the psychological intervention could benefit for reduce the failure rate. However, the present study has limitations. Firstly, the study sample source is single, further multi-center studies should be conducted to confirm the results. Secondly, the present study did not calculate the study size in advance and the sample size could expand in the further research. Moreover, as the present study may produce extra stress and pressure for the participants to be notified about the study before the sperm retrieval process, this could be limited for the study. Finally, most of the findings are based on quantitative self-report measures. Future researchers need to analyze the factor underlying the psychological stress, emotion regulation strategies, neuroticism, and sexual relationship on the levels of psychological and physical stress, which help to clarify the mechanisms of sexual dysfunctions.

## 5. Conclusion

This study demonstrated that social and psychological factors can influence the occurrence of TEF in men during IVF. The results indicate that psychological distress and neurotic personality were risk factors for the occurrence of TEF. Cognitive reappraisal and sexual relationship were protective factors in the prevention of ejaculation failure. The appropriated psychological intervention to male patients undergoing IVF treatment may help to reduce the psychological distress and improve sexual relationship and the occurrence of TEF.

## Data availability statement

The raw data supporting the conclusions of this article will be made available by the authors, without undue reservation.

## Ethics statement

The studies involving human participants were reviewed and approved by the Ethics Committee of Shandong University. The patients/participants provided their written informed consent to participate in this study.

## Author contributions

WW and LJY contributed to the conception and design of the study. WW and XTZ performed the statistical analysis and wrote the first draft of the manuscript. LXY, WW, and LJY wrote sections of the manuscript. All authors contributed to the article and approved the submitted version.

## Conflict of interest

The authors declare that the research was conducted in the absence of any commercial or financial relationships that could be construed as a potential conflict of interest.

## Publisher’s note

All claims expressed in this article are solely those of the authors and do not necessarily represent those of their affiliated organizations, or those of the publisher, the editors and the reviewers. Any product that may be evaluated in this article, or claim that may be made by its manufacturer, is not guaranteed or endorsed by the publisher.
